# Dietary Sources of Vitamin B-12 and Their Association with Vitamin B-12 Status Markers in Healthy Older Adults in the B-PROOF Study

**DOI:** 10.3390/nu7095364

**Published:** 2015-09-14

**Authors:** Elske M. Brouwer-Brolsma, Rosalie A. M. Dhonukshe-Rutten, Janneke P. van Wijngaarden, Nikita L. van der Zwaluw, Nathalie van der Velde, Lisette C. P. G. M. de Groot

**Affiliations:** 1 Division of Human Nutrition, Wageningen University, P.O. Box 17, Wageningen 6700 AA, The Netherlands; E-Mails: rosalie.dhonukshe-rutten@wur.nl (R.A.M.D.-R.); janneke.vanwijngaarden@gmail.com (J.P.V.W.); nikitavanderzwaluw@gmail.com (N.L.V.D.Z.); lisette.degroot@wur.nl (L.C.P.G.M.D.G.); 2 Erasmus MC, Department of Internal Medicine, P.O. Box 2040, Rotterdam 3000 CA, The Netherlands; E-Mail: n.vandervelde@erasmusmc.nl; 3 Section of Geriatric Medicine, Department of Internal Medicine, Academic Medical Center, P.O. Box 22700, Amsterdam 1100 DD, The Netherlands

**Keywords:** vitamin B-12 intake, serum vitamin B-12, dairy, milk, yoghurt, cheese, meat, fish and shellfish, eggs

## Abstract

Low vitamin B-12 concentrations are frequently observed among older adults. Malabsorption is hypothesized to be an important cause of vitamin B-12 inadequacy, but serum vitamin B-12 may also be differently affected by vitamin B-12 intake depending on food source. We examined associations between dietary sources of vitamin B-12 (meat, fish and shellfish, eggs, dairy) and serum vitamin B-12, using cross-sectional data of 600 Dutch community-dwelling adults (≥65 years). Dietary intake was assessed with a validated food frequency questionnaire. Vitamin B-12 concentrations were measured in serum. Associations were studied over tertiles of vitamin B-12 intake using *P* for trend, by calculating prevalence ratios (PRs), and splines. Whereas men had significantly higher vitamin B-12 intakes than women (median (25th–75th percentile): 4.18 (3.29–5.38) *versus* 3.47 (2.64–4.40) μg/day), serum vitamin B-12 did not differ between the two sexes (mean ± standard deviation (SD): 275 ± 104 pmol/L *versus* 290 ± 113 pmol/L). Higher intakes of dairy, meat, and fish and shellfish were significantly associated with higher serum vitamin B-12 concentrations, where meat and dairy—predominantly milk were the most potent sources. Egg intake did not significantly contribute to higher serum vitamin B-12 concentrations. Thus, dairy and meat were the most important contributors to serum vitamin B-12, followed by fish and shellfish.

## 1. Introduction

Vitamin B-12 deficiency is a common phenomenon among older adults all over the world [1]. As low vitamin B-12 concentrations have been associated with neuropsychiatric damage—even in cases of mild deficiency [2,3]—vitamin B-12 deficiency in old age can be considered a substantial public health problem. In most Western Societies, dietary vitamin B-12 intake is above dietary reference intakes [4,5]. Therefore, dietary insufficiency is not considered to be the main cause of vitamin B-12 deficiency in older adults.

Currently, malabsorption of food-bound vitamin B-12 is assumed to be the dominant cause of low circulating vitamin B-12 concentrations among older adults [1,3,6]. Absorption of food-bound vitamin B-12 is complex and requires several steps, including (1) release from its matrix in the acidic environment of the stomach; (2) binding to haptocorrin; (3) digestion of haptocorrin complex by pancreatic proteases, and subsequent release of vitamin B-12; which (4) is in turn bound to intrinsic factor; after which (5), intrinsic factor B-12 complex is absorbed in the ileum. Atrophic gastritis can result in an impaired release of vitamin B-12 from its carriers in food and as such disturb the above described process [1].

Atrophic gastritis may occur in up to 50% of older adults—where some Asian studies show even higher prevalences [7]—and has been associated with low circulating vitamin B-12 concentrations [8,9] and high circulating methylmalonic acid (MMA) concentrations [9]. Along with the fact that atrophic gastritis is considered to be an important factor contributing to vitamin B-12 deficiency, it is most important to gain better insight in the bioavailability of vitamin B-12 from different food sources. Particularly in cases of modest vitamin B-12 deficiency, targeted dietary recommendations may contribute to reversing low circulating vitamin B-12 concentrations.

An early study using data of the Framingham Offspring Study indicated that particularly use of supplements, fortified cereals, and milk were associated with higher plasma vitamin B-12 concentrations [10]; the potency of meat, poultry, and fish to increase vitamin B-12 concentrations seemed to be lower. In the Norwegian Hordaland Homocysteine Study, higher intakes of vitamin B-12 from dairy products, particularly milk and fish, were associated with higher plasma vitamin B-12 concentrations, whereas vitamin B-12 intakes obtained from meat or eggs intake were not [11]. According to dose-response associations for the different product groups, dairy intake resulted in the steepest increase in vitamin B-12 concentrations, suggesting that vitamin B-12 supplied by dairy product intake had the highest bioavailability [11].

To verify these results, we examined associations between various dietary vitamin B-12 sources—including meat and meat products, fish and shellfish, eggs, and dairy products—and serum vitamin B-12 in a Dutch population of older adults aged ≥65 years.

## 2. Experimental Section

### 2.1. Study Population

This cross-sectional study was performed using baseline data of the B-PROOF study. The B-PROOF study is a randomized, placebo-controlled, double blind intervention study investigating the effect of supplementation of folic acid (400 μg) and vitamin B-12 (500 μg) on the prevention of osteoporotic fractures in 2919 mildly hyperhomocysteinemic older adults ≥65 years. This large multi-center project was conducted in The Netherlands by a consortium from Erasmus MC (EMC, Rotterdam), VU University Medical Center (VUmc, Amsterdam), and Wageningen University (WU, Wageningen), with the latter acting as coordinator. Recruitment took place from August 2008 until March 2011. Participants were excluded if they used supplements or injections with high dose of B-vitamins during the past four months, were bedridden, incompetent to make own decisions, diagnosed with cancer (except certain types of skin cancer) in the past five years, had low (<12 μmol/L) or high (>50 μmol/L) plasma homocysteine levels, or renal dysfunction (serum creatinine levels >150 μmol/L). Vitamin B-12 intake from foods was only assessed in the Wageningen study population, where 600 participants provided reliable data on food intake and serum vitamin B-12. Further details on the design and methods of the B-PROOF study can be obtained from the design article [12]. The Medical Ethics Committee of Wageningen UR approved the study protocol and Medical Ethics Committees of VUmc and Erasmus MC confirmed local feasibility. All participants gave written informed consent. RCT trial registration: The B-PROOF study has been registered with ClinicalTrials.gov under identifier NCT00696514 since 9 June 2008.

### 2.2. Dietary Assessment

A Food Frequency Questionnaire (FFQ) containing 190 food items, specifically designed to assess intake of macronutrients, vitamin B-12, folate, vitamin D, and calcium, was used to estimate habitual dietary vitamin B-12 intake. The FFQ was administered once. The FFQ was developed by the dietetics group at the Division of Human Nutrition at Wageningen University and was validated for energy, fat, cholesterol, folate, and vitamin B-12 intake [13,14]. An item was added to the FFQ when it contributed to more than 0.1% of the intake of the nutrients where it was designed for. All items in the FFQ accounted for 90% of the total folate and vitamin B-12 intake according to Dutch National Food Consumption Survey data of 1998 [15]. The Dutch Food Composition database (NEVO) was used to calculate daily vitamin B-12 intake [16].

### 2.3. Biochemical Analyses

Blood samples were obtained from participants in the morning at the research center or at external locations in the living area of participants. Participants were in a fasted state, or had taken a light breakfast. To obtain plasma and serum a skilled nurse performed blood sampling of venous blood. For homocysteine determinations, blood collected in EDTA tubes were stored on ice water immediately after sampling, to prevent temperature- and time-dependent increases in plasma homocysteine [17,18]. Plasma homocysteine was measured in batches at the laboratory of Wageningen University using High-performance liquid chromatography (HPLC) (intra assay Coefficient of Variation (CV): 3.1%, inter assay CV: 5.9%) [19]. Serum vitamin B-12, folate, HoloTC, and methylmalonic acid (MMA) analyses were performed at the Laboratory of the Erasmus Medical Centre; all samples were analyzed at once when all samples were collected. Serum concentrations of vitamin B-12 and folate were determined by immunoelectrochemiluminescence assay (Elecsys 2010, Roche GmbH, Mannheim, Germany) (CV vitamin B-12 5.1% at 125 pmol/L and 2.9% at 753 pmol/L; CV folate: 5.9% at 5.7 nmol/L and 2.8% at 23.4 nmol/L) [20]. Holotranscobalamin (HoloTC) was determined by the AxSYM analyzer (Abbott, Wiesbaden, Germany) (CV < 8%) and serum concentrations of MMA were determined by modified gas chromatography-mass spectrometry (LC-MS/MS) (CV < 9%) [21]. Serum creatinine was measured with the enzymatic colorimetric Roche CREA plus assay (CV: 2%).

### 2.4. Covariates

Height was measured at baseline with a stadiometer to the nearest 0.1 cm. Weight was measured to the nearest 0.5 kg with a calibrated analogue scale. Body Mass Index (BMI) was calculated as weight/height^2^. Data on education level (primary, secondary or higher education), smoking status (non-smoker, current smoker, former smoker), physical activity (min/day) [22], and alcohol intake (light, moderate, excessive) were collected by means of questionnaires.

### 2.5. Data Analyses

Participant characteristics are reported as mean with standard deviation (SD), or percentages. Medians with interquartile range (25th–75th percentile) were used to report skewed variables. Differences between groups were analyzed using ANOVA in case of normally distributed continuous variables, Kruskal-Wallis in case of skewed continuous variables, and chi-square test in case of categorical variables. In order to compare serum vitamin B-12 concentrations across tertiles of vitamin B-12 intake from food items, analysis of covariance (ANCOVA) was performed in order to obtain adjusted means with 95% confidence intervals (95% CI). Compared food items included total vitamin B-12 intake, vitamin B-12 intake from meat, vitamin B-12 intake from fish and shellfish, vitamin B-12 intake from eggs, and vitamin B-12 intake from dairy. Furthermore, also subclasses of total dairy intake were examined, including vitamin B-12 intake from milk, vitamin B-12 intake from yogurt, and vitamin B-12 intake from cheese. *p*-for trend across tertiles of vitamin B-12 intake was calculated to assess a potentially statistical significant dose-response relationship between vitamin B-12 intake and serum vitamin B-12. Cox proportional hazards analysis with robust error variance was conducted to calculate Prevalence Ratios (PRs) for adequate serum vitamin B-12 concentrations (≥200 pmol/L) and normal serum vitamin B-12 and MMA concentrations (serum vitamin B-12 ≥200 pmol/L and methylmalonic acid <0.27 μmol/L) [11] by tertiles of vitamin B-12 intake from different dietary sources, using the lowest tertile as the reference group. By assigning a constant risk period to all participants in the study, the obtained hazard ratio can be considered a prevalence ratio (PR) [23]. This PR corresponds to the probability of having adequate serum vitamin B-12 concentrations or normal serum vitamin B-12 and MMA concentrations in participants in the middle and upper vitamin B-12 intake tertile, compared to participants in the lowest vitamin B-12 intake tertile. Analyses were adjusted for age, sex (model 1), BMI, education, alcohol intake, physical activity level, smoking, creatinine (model 2), total energy intake, and intake of vitamin B-12-containing food items other than the exposure for the specific model (model 3). Again, *P*-for trend across tertiles of vitamin B-12 intake was calculated to assess a potential statistically significant dose-response relationship of vitamin B-12 intake with the probability of having an adequate serum vitamin B-12 concentration or normal serum vitamin B-12 and MMA concentrations. Associations of vitamin B-12 intake (continuously) with the probability of having an adequate serum vitamin B-12 concentration (dichotomously) were furthermore investigated, using restricted cubic spline regression conducted with three knots located at 1st decile of intake, 5th decile of intake, and 9th decile of intake. All analyses were performed using the statistical package SAS, version 9.2 (SAS Institute Inc., Cary, NC, USA). Unless stated otherwise, a two-sided p-value of ≤0.05 was considered to be statistically significant.

## 3. Results

Population characteristics are shown in Table 1. Participants were on average 72 years old. When comparing characteristics of men and women, men had significantly lower BMI (mean ± SD: 26.2 ± 3.0 *versus* 27.3 ± 4.3 kg/m^2^), were less likely to be non-smoker (20% *versus* 46%), less active (mean ± SD: 139 ± 91 *versus* 161 ± 109 min per day), more likely to have moderate alcohol intake (38% *versus* 26%), have lower HoloTC concentrations (median (25th–75th percentile): 57 (43–76) *versus* 63 (48–87) pmol/L), and higher creatinine concentrations (mean ± SD: 91 ± 17 *versus* 75 ± 14 μmol/L). When comparing participants with an adequate serum vitamin B-12 concentration (≥200 pmol/L) with those with an inadequate serum vitamin B-12 concentration (<200 pmol/L), those with an adequate concentration appeared to be significantly younger (mean ± SD: 72.0 ± 5.2 *versus* 73.0 ± 5.8 years) and to have higher creatinine concentrations (mean ± SD: 85 ± 18 *versus* 82 ± 18 µmol/L). Moreover, as may be expected, those with an adequate serum vitamin B-12 concentration had significantly lower serum MMA and plasma homocysteine concentrations, and higher serum HoloTC and folate concentrations.

Table 2 provides an overview of the differences in dietary intakes between men and women, between those with an adequate (≥200 pmol/L) and inadequate serum vitamin B-12 concentration (<200 pmol/L), and between those with normal *versus* impaired serum vitamin B-12 and MMA concentrations (serum vitamin B-12 <200 pmol/L with MMA concentration >0.27 μmol/L). When comparing participants with an adequate serum vitamin B-12 concentration with those with an inadequate serum vitamin B-12 concentration, significantly lower dietary intakes were observed for amongst others protein (mean ± SD: 15 ± 2 En% *versus* 14 ± 2 En%), vitamin B-12 from meat (median (25th–75th percentile): 1.20 (0.85–1.77) *versus* 1.08 (0.62–1.74) μg/day), vitamin B-12 from fish and shellfish (median (25th–75th percentile): 0.54 (0.26–1.08) *versus* 0.34 (0.13–0.93) μg/day), vitamin B-12 from dairy (median (25th–75th percentile): 1.30 (0.90–1.67) *versus* 1.07 (0.77–1.58) μg/day), and vitamin B-12 from milk (median (25th–75th percentile): 0.52 (0.19–0.93) *versus* 0.36 (0.09–0.71) μg/day). In addition, when comparing the food groups that contribute to the dietary vitamin B-12 intake, dairy (e.g., for men, median (25th–75th percentile): 1.32 (0.89–1.71) μg/day) and meat products (e.g., for men, median (25th–75th percentile): 1.31 (0.91–1.89) μg/day) appeared to be the most important contributors to vitamin B-12 intake, followed by fish and shellfish (e.g., for men, median (25th–75th percentile): 0.61 (0.26–1.20) μg/day), and eggs (e.g., for men, median (25th–75th percentile): 0.16 (0.08–0.24) μg/day.

**Table 1:** Baseline characteristics of the participants of the B-PROOF Study (*n* = 600) *.

	Men	Women	Serum Vitamin B-12 < 200 pmol/L	Serum Vitamin B-12 ≥ 200 pmol/L	Impaired Serum Vitamin B-12 and MMA **	Normal Serum Vitamin B-12 and MMA
*N*	348	252	134	466	68	531
Men *n* (%)	348 (100)	0	87 (65)	261 (56)	46 (68)	302 (57)
Age, years	72.1 ± 5.1	72.4 ± 5.7	73.0 ± 5.8	72.0 ± 5.2 ^#^	73.8 ± 5.7	72 ± 5 ^#^
Body Mass Index, kg/m^2^	26.2 ± 3.0	27.3 ± 4.3 ^#^	27.0 ± 4.0	26.9 ± 3.5	26.4 ± 3.9	27.0 ± 3.5
Smoking, *n* (%)						
Non-smoker	70 (20)	115 (46) ^#^	35 (26)	150 (32)	13 (19)	172 (32)
Smoker	41 (12)	21 (8)	17 (13)	45 (10)	10 (15)	52 (10)
Former smoker	237 (68)	116 (46)	82 (61)	271 (58)	45 (66)	307 (58)
Physical activity, kcal/day	139 ± 91	161 ± 109 ^#^	158 ± 102	145 ± 98	139 ± 69	149 ± 102
Education, *n* (%)						
Primary	112 (32)	142 (56) ^#^	57 (42)	197 (42)	36 (53)	218 (41)
Secondary	97 (28)	48 (19)	36 (27)	109 (24)	14 (21)	131 (25)
Higher	139 (40)	62 (25)	41 (31)	160 (34)	18 (26)	182 (34)
Alcohol intake, *n* (%)						
Light	203 (58)	181 (72) ^#^	87 (65)	297 (64)	47 (69)	336 (63) ^#^
Moderate	133 (38)	65 (26)	43 (32)	155 (33)	17 (25)	181 (34)
Excessive	12 (4)	6 (2)	4 (3)	14 (3)	4 (6)	14 (3)
Serum vitamin B-12, pmol/L	275 ± 104	290 ± 113	163 ± 29	315 ± 98 ^#^	157 ± 30	297 ± 104 ^#^
Serum methylmalonic acid, µmol/L (*n* = 1 missing)	0.23 (0.19–0.30)	0.24 (0.19–0.41)	0.28 (0.22–0.39)	0.22 (0.18–0.28) ^#^	0.39 (0.31–0.59)	0.22 (0.18–0.27) ^#^
Serum HoloTC, pmol/L (*n* = 1 missing)	57 (43–76)	63 (48–87) ^#^	39 (29–52)	67 (52–86) ^#^	35 (26–44)	64 (49–82) ^#^
Plasma homocysteine, μmol/L	15.2 ± 3.3	14.9 ± 3.2	16.4 ± 4.2	14.7 ± 2.8 ^#^	17.4 ± 4.8	14.8 ± 2.9 ^#^
Serum folate, μg/day (*n* = 9 missing)	19.0 ± 6.9	19.4 ± 6.8	17.6 ± 6.2	19.5 ± 7.0 ^#^	17.8 ± 6.2	19.2 ± 6.9
Serum creatinine, μmol/L (*n* = 1 missing)	91 ± 17	75 ± 14 ^#^	82 ± 18	85 ± 18 ^#^	83 ± 19	84 ± 18

* Differences between groups were analyzed using ANOVA in case of normally distributed continuous variables, Kruskal-Wallis in case of skewed continuous variables, and chi-square test in case of categorical variables; values are presented as mean ± SD, or medians (25th and 75th percentile); ** Vitamin B-12 < 200 pmol/L with MMA > 0.27 μmol/L; as there is one missing for MMA, this column applies to 599 participants; ^#^ Groups significantly differed *p* ≤ 0.05.

**Table 2:** Dietary intakes of the participants of the B-PROOF Study (*n* = 600) *.

	Men	Women	Serum Vitamin B-12 <200 pmol/L	Serum Vitamin B-12 ≥200 pmol/L	Impaired Serum Vitamin B-12 and MMA **	Normal Serum Vitamin B-12 and MMA
*N*	348	252	134	466	68	531
Energy intake, kcal/day	2170 ± 484	1778 ± 350 ^#^	2058 ± 554	1990 ± 448	2075 ± 590	1998 ± 457
Fat, En%	36 ± 6	36 ± 6	36 ± 6	36 ± 6	37 ± 7	36 ± 6
Protein, En%	15 ± 2	16 ± 2 ^#^	14 ± 2	15 ± 2 ^#^	14 ± 3	15 ± 2 ^#^
Carbohydrates, En%	44 ± 6	44 ± 7	44 ± 7	44 ± 6	44 ± 8	44 ± 6
Fibre, gram/day	25 ± 7	23 ± 6 ^#^	24 ± 7	24 ± 6	24 ± 8	24 ± 6
Folate, μg/day	199 ± 56	181 ± 48 ^#^	184 ± 53	194 ± 54	182 ± 52	193 ± 54
Total vitamin B-12, μg/day	4.18 (3.29–5.38)	3.47 (2.64–4.40) ^#^	3.56 (2.54–4.61)	3.92 (3.10–5.17) ^#^	3.47 (2.59–4.29)	3.92 (3.00–5.17) ^#^
Vitamin B-12 from foods, μg/day	4.50 (3.18–5.17)	3.41 (2.62–4.31) ^#^	3.52 (2.43–4.61)	3.80 (3.00–5.01) ^#^	3.38 (2.59–4.29)	3.80 (2.94–5.00) ^#^
Vitamin B-12 from supplements, μg/day	0 (0–0)	0 (0–0)	0 (0–0)	0 (0–0)	0 (0–0)	0 (0–0)
Vitamin B-12 from meat, μg/day	1.31 (0.91–1.89)	1.01 (0.69–1.58) ^#^	1.08 (0.62–1.74)	1.20 (0.85–1.77) ^#^	1.11 (0.62–1.90)	1.19 (0.84–1.74)
Vitamin B-12 from fish and shellfish, μg/day	0.61 (0.26–1.20)	0.44 (0.19–0.82) ^#^	0.34 (0.13–0.93)	0.54 (0.26–1.08) ^#^	0.30 (0.09–0.65)	0.53 (0.25–1.12) ^#^
Vitamin B-12 from eggs, μg/day	0.16 (0.08–0.24)	0.16 (0.08–0.16)	0.16 (0.08–0.24)	0.16 (0.08–0.16)	0.16 (0.06–0.24)	0.16 (0.08–0.16)
Vitamin B-12 from dairy, μg/day	1.32 (0.89–1.71)	1.17 (0.83–1.56) ^#^	1.07 (0.77–1.58)	1.30 (0.90–1.67) ^#^	1.03 (0.73–1.58)	1.28 (0.88–1.67) ^#^
Vitamin B-12 from milk, μg/day	0.57 (0.22–1.00)	0.35 (0.10–0.69) ^#^	0.36 (0.09–0.71)	0.52 (0.19–0.93) ^#^	0.34 (0.06–0.65)	0.50 (0.17–0.90) ^#^
Vitamin B-12 from yogurt, μg/day	0.37 (0.08–0.56)	0.37 (0.13–0.56)	0.39 (0.13–0.55)	0.36 (0.09–0.56)	0.35 (0.02–0.55)	0.36 (0.12–0.56)
Vitamin B-12 from cheese, μg/day	0.17 (0.09–0.27)	0.20 (0.11–0.33) ^#^	0.17 (0.09–0.28)	0.18 (0.10–0.29)	0.16 (0.07–0.28)	0.18 (0.10–0.29)

* Comparisons between groups were analyzed using ANOVA in case of continuous variables and chi-square test in case of categorical variables; values are presented as mean ± SD, or medians (25th and 75th percentile); ** vitamin B-12 < 200 pmol/L with MMA > 0.27 μmol/L; as there is one missing for MMA, this column applies to 599 participants; ^#^ Groups significantly differed *p* ≤ 0.05.

Table 3 shows adjusted means of serum vitamin B-12 concentrations according to tertiles of vitamin B-12 intake from the different dietary sources, indicating that higher intakes of vitamin B-12 from meat, fish and shellfish, and dairy were significantly associated with higher serum vitamin B-12 concentrations, whereas vitamin B-12 as obtained by the intake of eggs was not. Although vitamin B-12 from dairy was significantly associated with serum vitamin B-12 concentrations, none of the specific dairy food groups had a significant impact on vitamin B-12 when explored individually. Nevertheless, given the borderline non-significant finding for milk (*i.e.*, T1: 256 (220; 292), T2: 264 (229; 300), T3: 283 (246; 319), *P* for trend: 0.06), it seems that the association between dairy and serum vitamin B-12 is largely explained by the intake of this product group. Vitamin B-12 intake per tertile of meat and dairy were almost comparable, as were the adjusted mean serum vitamin B-12 concentrations across tertiles, suggesting that there are no large differences in bioavailability between these two food groups.

Results in Table 4 confirm the findings in Table 3. Namely, the probability that participants in the upper tertile of vitamin B-12 intake as obtained by meat intake (≥1.52 μg/day) have an adequate serum vitamin B-12 concentration (PR, 95% CI: 1.22, 1.08–1.37) is comparable to the probability that participants in the upper tertile of vitamin B-12 intake as obtained by dairy intake (≥1.49 μg/day) have an adequate serum vitamin B-12 concentration (PR, 95% CI: 1.24, 1.10–1.39). For fish and shellfish intake, the probability of having an adequate serum vitamin B-12 concentration was 16% in the upper intake tertile (≥0.77 μg/day) when compared to the lowest tertile (PR, 95%: 1.16, 1.04–1.30). The borderline non-significant results for milk in Table 3, did appear to be statistically significant when investigated in relation to having an adequate serum vitamin B-12 concentration (PR, 95% CI for T1:1.0; T2: 1.12, 1.00–1.25; T3: 1.19, 1.06–1.33; *P* for trend: 0.004). For all food groups under study, differences in results between model 2 and model 3 were predominantly driven by additional adjustment for total energy intake.

**Table 3:** Adjusted means and 95% CIs for serum vitamin B-12 concentrations by tertiles of vitamin B-12 intake.

	T1	T2	T3	*P* for Trend
Total vitamin B-12 intake, μg/day	≤3.19	3.20–4.40	≥4.41	
Serum B-12, pmol/L	250 (214; 286)	273 (237; 310)	287 (250; 324)	0.009
Vitamin B-12 intake from meat, μg/day	≤0.92	0.93–1.51	≥1.52	
Serum B-12, pmol/L	247 (211; 284)	275 (240; 311)	290 (253; 327)	0.001
Vitamin B-12 intake from fish and shellfish, μg/day	≤0.31	0.32–0.76	≥0.77	
Serum B-12, pmol/L	254 (218; 290)	274 (238; 310)	281 (244; 317)	0.04
Vitamin B-12 intake from eggs, μg/day	≤0.078	0.079–0.157	≥0.158	
Serum B-12, pmol/L	265 (229; 301)	277 (241; 312)	265 (228; 302)	0.43
Vitamin B-12 intake from dairy products, μg/day	≤0.96	0.97–1.48	≥1.49	
Serum B-12, pmol/L	249 (214; 284)	286 (250; 323)	287 (250; 324)	0.0006

Means were adjusted for age, sex, BMI, education, alcohol intake, physical activity level, smoking, creatinine, total energy intake, and intake of other vitamin B-12-containing food items, and calculated by ANCOVA.

**Table 4:** Prevalence ratios and 95% CIs of participants with serum vitamin B-12-concentations ≥ 200 pmol/L *vs* <200 pmol/L or normal serum vitamin B-12 and MMA *vs.* impaired serum vitamin B-12 and MMA (vitamin B-12 < 200 pmol/L and methylmalonic acid >0.27 μmol/L) by tertiles of vitamin B-12 intake from different dietary sources.

	Probability of Having Serum Vitamin B-12 ≥200 pmol/L			Probability of Having Normal Serum Vitamin B-12 and MMA		
Dietary vitamin B-12 intake from food items (μg/day)	Model 1	Model 2	Model 3	Model 1	Model 2	Model 3
Total vitamin B-12 intake						
≤3.19	1 (ref)*	1 (ref)	1 (ref)	1 (ref)	1 (ref)	1 (ref)
3.20–4.40	1.16 (1.04–1.30)	1.16 (1.05–1.30)	1.19 (1.07–1.33)	1.05 (0.97–1.13)	1.05 (0.97–1.13)	1.07 (0.99–1.15)
≥4.41	1.14 (1.02–1.28)	1.14 (1.02–1.28)	1.20 (1.06–1.35)	1.11 (1.04–1.19)	1.10 (1.03–1.18)	1.14 (1.04–1.24)
*P* for trend	0.04	0.03	0.007	0.003	0.005	0.003
Vitamin B-12 intake from meat						
≤0.92	1 (ref)	1 (ref)	1 (ref)	1 (ref)	1 (ref)	1 (ref)
0.93–1.51	1.13 (1.01–1.26)	1.13 (1.01–1.26)	1.17 (1.04–1.31)	1.06 (0.99–1.14)	1.06 (0.99–1.14)	1.09 (1.01–1.17)
≥1.52	1.14 (1.02–1.28)	1.15 (1.02–1.28)	1.22 (1.08–1.37)	1.05 (0.97–1.13)	1.04 (0.97–1.12)	1.07 (0.99–1.17)
*P* for trend	0.03	0.02	0.02	0.30	0.37	0.13
Vitamin B-12 intake from fish and shellfish						
≤0.31	1 (ref)	1 (ref)	1 (ref)	1 (ref)	1 (ref)	1 (ref)
0.32-0.76	1.18 (1.06–1.31)	1.17 (1.05–1.31)	1.17 (1.05–1.30)	1.12 (1.03–1.20)	1.11 (1.03–1.19)	1.10 (1.02–1.18)
≥0.77	1.15 (1.03–1.29)	1.15 (1.03–1.29)	1.16 (1.04–1.30)	1.14 (1.06–1.23)	1.13 (1.04–1.21)	1.13 (1.05–1.23)
*P* for trend	0.03	0.04	0.02	0.01	0.004	0.003
Vitamin B-12 intake from eggs						
≤0.078	1 (ref)	1 (ref)	1 (ref)	1 (ref)	1 (ref)	1 (ref)
0.079–0.157	1.10 (1.00–1.21)	1.11 (1.00–1.22)	1.10 (0.99–1.21)	1.04 (0.97-1.10)	1.04 (0.98–1.11)	1.04 (0.97–1.11)
≥0.158	1.04 (0.92–1.17)	1.06 (0.94–1.19)	1.05 (0.93–1.18)	1.00 (0.93-1.08)	1.01 (0.94–1.09)	1.01 (0.93–1.09)
*P* for trend	0.51	0.35	0.43	0.94	0.76	0.87
Vitamin B-12 intake from dairy products						
≤0.96	1 (ref)	1 (ref)	1 (ref)	1 (ref)	1 (ref)	1 (ref)
0.97–1.48	1.19 (1.06–1.33)	1.17 (1.05–1.31)	1.20 (1.08–1.34)	1.10 (1.03–1.19)	1.09 (1.01–1.17)	1.10 (1.02–1.19)
≥1.49	1.18 (1.06–1.33)	1.18 (1.06–1.33)	1.24 (1.10–1.39)	1.10 (1.02–1.18)	1.09 (1.01–1.18)	1.11 (1.02–1.21)
*P* for trend	0.006	0.005	0.0007	0.03	0.04	0.02

Model 1 was adjusted for age and sex; Model 2 was adjusted for age, sex, BMI, education, alcohol intake, physical activity level, smoking, and creatinine; Model 3 was adjusted for age, sex, BMI, education, alcohol intake, physical activity level, smoking, creatinine, total energy intake, and intake of other vitamin B-12-containing food items; ref is the reference group to which the other groups or categories are compared to.

**Figure 1:**
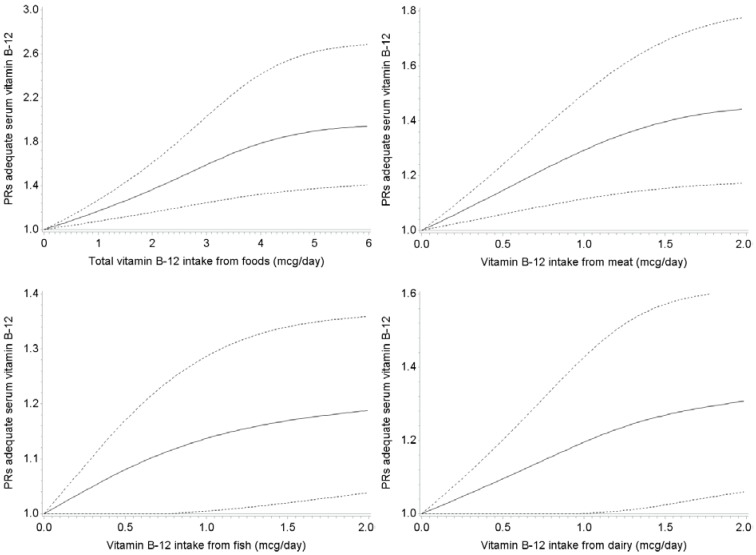
Associations between vitamin B-12-intake from different food sources with serum vitamin B-12-concentations ≥ 200 pmol/L, obtained by spline regression with three knots located at 1st, 5th, and 9th decile. Dotted lines represent 95% confidence intervals. Prevalence ratios were adjusted for age, sex, BMI, education, alcohol intake, physical activity level, smoking, creatinine, total energy intake, and intake of other vitamin B-12-containing food items. *P* for non-linearity was 0.003 for total vitamin B-12 intake; 0.002 for vitamin B-12 intake from meat; 0.16 for vitamin B-12 intake from fish and shellfish; and 0.34 for vitamin B-12 intake from dairy. *P* for linearity was 0.19 for vitamin B-12 intake from eggs; 0.06 for vitamin B-12 intake from milk; 0.94 for vitamin B-12 intake from yogurt; 0.44 for vitamin B-12 intake from cheese (data not shown).

The dose-response association was further examined by visualizing the associations between vitamin B-12 intake from different dietary sources and the probability of having adequate an serum vitamin B-12 concentration by spline regression analyses, where vitamin B-12 intake was modelled continuously (Figure 1). In addition to previous results, these graphs furthermore suggest that—at an intake level of 1 μg/day—dairy and meat seem to be somewhat more potent in reducing the probability of having an inadequate serum vitamin B-12 concentration than fish and shellfish in this population.

## 4. Discussion

In this study, higher dietary vitamin B-12 intake was significantly associated with higher serum vitamin B-12 concentrations. Dairy and meat were the most important suppliers of dietary vitamin B-12 in this population, followed by fish and shellfish. Vitamin B-12 intake resulting from egg consumption was relatively low and did not significantly associate with increasing serum vitamin B-12 concentrations. When exploring the intake of dairy products in more detail, specifically by studying milk, yogurt, and cheese intake individually, milk intake was associated with the highest serum vitamin B-12 concentrations.

Before extending these findings to the results of previous studies and speculating on the potential practical application of these findings, first some limitations and strengths need to be discussed. First of all, sample size of the current study was relatively small compared to the studies by Tucker and colleagues (2000) [10] and Vogiatzoglou and colleagues (2009) [11]. However, based on the significant associations observed in our study, power does not seem to be an issue and therefore results of this study do contribute to the current scientific evidence in this field as data on bioavailability of vitamin B-12 from different food sources is very limited. Secondly, we explored differences in bioavailability from different vitamin B-12 food sources. It should be kept in mind though, that whereas vitamin B-12 intakes obtained from dairy and meat were in the same range, vitamin B-12 intakes from fish and shellfish, eggs, and individual dairy subgroups were lower. This limits comparability of these food sources to for instance total dairy and meat intakes. Thirdly, this study was conducted in a population with mildly elevated homocysteine concentrations, which may have resulted from lower vitamin B-12 intakes in this population when compared to a general population of older adults. When comparing our vitamin B-12 intake data with vitamin B-12 intake data as obtained by the recent Dutch Food Consumption Survey 2010–2012, median vitamin B-12 intake in the B-PROOF population indeed appears to be slightly lower than vitamin B-12 intake in the general older population. To be more specific, according to the Dutch Food Consumption Survey, which assessed vitamin B-12 intake in a population that is considered representative for the total older Dutch population, median vitamin B-12 intake from foods in men was 5.0 (25th–75th percentile: 3.9–6.4) μg/day and in women this was 4.3 (3.2–5.2) μg/day. In our population median (25th–75th percentile) vitamin B-12 intake was 4.5 (3.2–5.2) μg/day in men and 3.4 (2.6–4.3) μg/day in women. Fourthly, although diabetes has been associated with lower serum vitamin B-12 concentrations, diabetes patients were not excluded in this study. However, self-reported diabetes was assessed in 377 participants of the B-PROOF population, where no distinction was made between type I and type II diabetes. Additional analyses in this subgroup did not indicate that adjustment for diabetes resulted in substantially different outcomes; nor did sensitivity analyses in which participants reporting diabetes (*n* = 27 of *n* = 377) were excluded. Strengths of this study include the use of an FFQ that was validated for macronutrients as well as vitamin B-12 and folate intake, and the possibility to include important potential relevant covariates.

Currently, the Dutch Health Council recommends older adults meet a vitamin B-12 intake of 2.8 μg/day [24], which is met by the majority of the participants of the B-PROOF study. Spline regression suggested that the impact of total vitamin B-12 intake from foods to ensure adequate serum vitamin B-12 concentrations in our population levelled off somewhere around an intake of 6 μg/day. In a population of 98 Danish postmenopausal women, curves for several vitamin B-12 related variables—including circulating vitamin B-12 concentrations, HoloTC, MMA, and total homocysteine—also levelled off at a daily vitamin B-12 intake of 6 μg/day [25]. In the Framingham Offspring study [10] and Hordaland Homocysteine Study [11] this plateau was located around 10 μg/day.

When studying specific vitamin B-12 rich foods, we observed that dairy and meat product intake were significantly associated with higher serum vitamin B-12 concentrations. Dose-response analyses by means of spline regression subsequently suggested that both food groups possessed an about equal potency to raise vitamin B-12 concentrations. More specifically, at an intake level of 1 μg vitamin B-12/day as obtained by meat, participants were 30% more likely to have adequate serum vitamin B-12 concentrations compared to those consuming 0 μg vitamin B-12/day as obtained by meat. For dairy, this probability was 20% higher for those consuming 1 μg vitamin B-12/day. Of the dairy subclasses, milk seemed to be the most important component of dairy intake in order to raise serum vitamin B-12 concentrations. Our findings with respect to meat are in contrast to the findings in the Hordaland Homocysteine Study and the Framingham Offspring Study [10,11], which did not observe a significant impact of meat on circulating vitamin B-12 concentrations while median vitamin B-12 intake by meat intake was comparable (*i.e.*, ±1.3 μg/day). It may be speculated that this discrepancy with respect to the impact of meat intake in raising vitamin B-12 concentrations between our study and Hordaland Homocysteine Study and the Framingham Offspring Study relates to the overall higher vitamin B-12 intakes and higher intakes of fish and shellfish in the Hordaland Homocysteine Study and the Framingham Offspring Study compared to our study and the suggested maximal load of vitamin B-12 intestinal absorption system per meal (*i.e.*, ±1.5–2.0 μg/meal). With respect to dairy intake, our findings are in line with these previous studies, where the Framingham Offspring Study also suggested a significant impact of dairy—particularly milk as did the Hordaland Homocysteine Study [10,11]. Although the impact of fish and shellfish on vitamin B-12 concentrations in our population was smaller than the impact of meat and dairy, higher fish and shellfish intake was significantly associated with higher vitamin B-12 concentrations, indicating that persons with a vitamin B-12 intake of 1 μg/day as obtained by fish and shellfish had a 13% higher probability of having an adequate serum vitamin B-12 concentration compared to those not consuming any fish or shellfish. The fact that the impact of fish and shellfish intake and serum vitamin B-12 in our population was less strong than the impact of dairy and meat, while this was not the case in for instance the Hordaland Homocysteine Study, may relate to the fact that the overall vitamin B-12 intake from fish and shellfish in our population (e.g., for men, median (25th–75th percentile): 0.61 (0.26–1.20) μg/day) was substantially lower than the overall fish and shellfish intake in the Hordaland Homocysteine Study (e.g., for men aged 71–74 years old, median (25th–75th percentile): 3.4 (2.0–5.3) μg/day) [11]. Finally, the null findings in this study with respect to vitamin B-12 as obtained by egg intake are in line with the findings in the Hordaland Homocysteine Study [11]; in both studies vitamin B-12 intake as obtained by eggs was low and the range was narrow, which may explain the low impact on circulating vitamin B-12 concentrations of this product group.

## 5. Conclusions

Our data suggest that vitamin B-12 as obtained from dairy, meat, and fish and shellfish substantially contribute to raise vitamin B-12 concentrations in a Dutch population of older adults, where dairy—particularly milk and meat seem to be the most potent sources in order to prevent inadequate serum vitamin B-12 concentrations. Next to determining—and if possible treating—the cause of the vitamin B-12 deficiency, targeted recommendations focusing on these food groups may help to counteract low vitamin B-12 concentrations, especially in cases of modest vitamin B-12 deficiency. In cases of more severe vitamin B-12 deficiency other strategies may be warranted, including for instance recommending high dose oral vitamin B-12 supplements or vitamin B-12 injections.
